# Subjective workload, professional environment and job satisfaction of anesthesia and intensive care nurses: a cross-sectional survey study

**DOI:** 10.3389/fpubh.2025.1663535

**Published:** 2025-09-02

**Authors:** Vilma Žydžiūnaitė, Viktorija Mickevičiūtė

**Affiliations:** Department of Nursing, Faculty of Health Sciences, Klaipėda University, Klaipėda, Lithuania

**Keywords:** anesthesia/intensive care nurse, cross-sectional study, job satisfaction, professional environment, subjective workload

## Abstract

**Background:**

There are studies that reveal the relationship between workload and job satisfaction, studies that would examine workload, professional environment, and job satisfaction separately, but there is still lack of the empirical evidence which proves various combinations between all these mentioned variables internationally within the anesthesia and intensive care nursing community.

**Aim:**

This study aimed to analyze the relationship between anesthesia and intensive care nurses’ subjective workload, professional environment, and job satisfaction.

**Methods:**

149 anesthesia and intensive care nurses working in the clinical hospitals of one county of Lithuania participated in the study. NASA Task Load Index (NASA-TLX), The Practice Environment Scale of the Nursing Work Index, and The Questionnaire of the Relation Between Job Satisfaction and Workload were used for data collection. IBM Statistical Package for Social Sciences Statistics for Windows, version 29.0 was used for data analysis.

**Results:**

Anesthesia and intensive care nurses experience above average or high subjective workload and rate their professional environment and job satisfaction as average. Nurses who experience higher subjective workload experience lower job satisfaction. Similarly, nurses who work in a more favorable professional environment experience lower workload and higher job satisfaction. The highest job satisfaction is among anesthesia and intensive care nurses who experience average workload and rate their professional environment positively. With high workload and unfavorable professional environment, anesthesia and intensive care nurses’ job satisfaction decreases.

**Conclusion:**

There is a relationship between anesthesia and intensive care nurses’ workload, professional environment and job satisfaction, regardless of geographical boundaries or different locations, the structure of the healthcare system, and these factors constantly affect each other. Subjective workload, work environment, and job satisfaction of anesthesia and intensive care nurses are interrelated and influence each other. Nurses who work in an unfavorable environment without managerial support, collaboration, and teamwork experience higher workload and lower job satisfaction. This means that job satisfaction decreases when there is a high workload and an unfavorable work environment.

## 1 Introduction

Nurses’ workload and professional environment are among the most important factors determining their job satisfaction (1). Nurses often face excessive workload, which negatively affects their mental and physical health and job satisfaction (1–4). A study conducted in Indonesia with 96 nurses, showed that 60.4% of respondents experienced high psychological workload, and a further 13.5% – very high. These data indicate that a significant proportion of nurses face excessive workload (2). Similar results were obtained in a study conducted in Italy, which included 334 nurses from three hospitals. Results revealed that nurses’ subjective workload was high, consisting of physical, mental and emotional components. The mental and emotional strain was particularly high, while physical strain was moderate (3). A study conducted in the Republic of Korea involving 32 nurses revealed that nurses experience high workload (4). Researchers from Ethiopia conducted a study with 407 nurses from five hospitals and found that 54% of them considered their working conditions to be inadequate (5). A quantitative cross-sectional study conducted in Mexico found that out of 510 nurses, one-third reported a poor or unfavorable professional environment (6).

Job satisfaction is related to workload and professional environment. A systematic review made by researchers from Italy showed that nurses’ job satisfaction is assessed from low to moderate, with the greatest dissatisfaction being due to poor professional environment and high workload (7). A study from Indonesia with 392 general practice nurses confirmed the relationship between workload, professional environment, and job satisfaction. It was found that insufficient staffing and lack of medical equipment increase workload, which negatively affects the quality of nurses’ work and job satisfaction (8).

Nurse shortages and increasing workloads are a global problem that threatens the quality and safety of healthcare services (9). Understaffing and high stress contribute to nurses’ job dissatisfaction (10–12). High workloads, poor professional environments, and low job satisfaction negatively affect not only nurses’ well-being but also patient safety, increasing the risk of medical errors, nosocomial infections, and mortality. As a result, nurses experience high levels of stress, which can lead to burnout and reduce the quality of nursing services (11). Researchers have highlighted the importance of this issue, revealing the links between nurses’ workload, professional environment and job satisfaction (13–15). The conditions of patients in anesthesia and intensive care units, which require specialist nursing skills, may change rapidly, so nurses must not only implement routine nursing plans, but also constantly monitor patients’ conditions and respond quickly to changes in order to save their lives (16, 17).

Anesthesia and intensive care nurses experience higher workloads than nurses in other units (18, 19). High workload and staffing shortages cause stress, burnout, and increased turnover in nurses, which reduces job satisfaction (20, 21). Although there are studies that reveal the relationship between workload and job satisfaction (11–15), studies that would examine workload, professional environment, and job satisfaction together lack the empirical evidence nationally and internationally. There is lack of studies that would reveal these relationships in the anesthesia and intensive care nursing community.

A large number of researchers emphasize that in order to improve the quality of nursing services and nurses’ job satisfaction, it is necessary to take into account the workload of nurses and the professional environment (22). Nurse shortages and burnout are increasingly common problems in the healthcare sector (23–25). Research on the relationship between workload, professional environment and job satisfaction can provide new insights into how working conditions and nurses’ well-being can be improved. It is known that subjective workload, professional environment have an important impact on job satisfaction of nurses. Studies have concerned job satisfaction among critical care nurses (26, 27). Not as many have focused on anesthesia and intensive care nurses.

The novelty in researching subjective workload, professional environment, and job satisfaction of anesthesia and intensive care nurses lies in understanding how these factors interact to impact nurse well-being and potentially influence patient care quality. Specifically, exploring the unique pressures and stressors within these specialized settings, and how they correlate with nurses’ job satisfaction and intent to stay, can provide valuable insights for improving nurse retention and optimizing patient outcomes.

The aim of the study is to analyze the relationship between anesthesia and intensive care nurses’ subjective workload, professional environment, and job satisfaction.

## 2 Materials and methods

### 2.1 Design

The study used a cross-sectional design. This design is valuable in nursing research, because it is used to determine prevalence, identify associations between variables, and inform nursing practice and generate directions and/or hypotheses for further research (28).

### 2.2 Sample

The study population consists of anesthesia and intensive care nurses working in hospitals in Klaipėda, the only Lithuanian county located on the Baltic Sea coast. Klaipėda county includes 7 municipalities covering nine cities and 1.001 rural residential areas. There is a total of 13 hospitals with anesthesia and intensive care departments, where the survey was conducted.

According to the data provided in the personnel statistical report by the Institute of Hygiene (29) of Lithuania, 227 anesthesia and intensive care nurses worked in Klaipėda County.

There are the following five nursing specializations in Lithuania: operating room nursing, mental health nursing, anesthesia and intensive care nursing, emergency medical care nursing, and community nursing. This study focuses on nurses who have acquired anesthesia and intensive care specialization and work in the practical field of nursing in this specialization in clinical hospitals. These nurses work with physician anesthesiologists during surgeries, administer anesthesia medications, and provide assistance in critical and life-threatening conditions, monitoring, maintaining, and stabilizing vital functions of patients.

The sample was formed based on inclusion and exclusion criteria:

- Inclusion criteria: practicing anesthesia and intensive care nursing in an inpatient healthcare institutions in Klaipėda county, having acquired basic nursing education and specialization in anesthesia and intensive care nursing.
- Exclusion criteria: lack of a certificate of specialization in anesthesia and intensive care nursing, not working as a nurse, working in another nursing specialization (not anesthesia and intensive care), working in other (not Klaipeda) municipal hospitals.

The sample size of the study population was calculated according to the Paniott formula, in which n – sample size; *N* – general population size; *Δ* = statistical error rate (Δ = 0.05) (30, 31):


$$n=\frac{1}{\Delta^{2}+\frac{1}{N}}$$

Based on Paniott’s formula and applying a 95% confidence level and a 5% margin of error, it was calculated that the non-probability-purposive sample should consist of at least 145 respondent nurses. The study participants were selected using a non-probability-purposive sampling method.

175 questionnaires were distributed, of which 149 correctly completed questionnaires were returned. In total 149 anesthesia and intensive care nurses working in Klaipėda county hospitals participated in the study (see Table 1).

**Table 1:** Distribution of study participants by sociodemographic indicators (*N* = 149).

Attribute		No. of respondents	%
Gender	Man	20	13.4
	Woman	129	86.6
Age	20–30 years old	40	26.8
	31–40 years old	47	31.5
	> 41 years old	62	41.6
Education	Diploma Nurse	46	30.9
	College level Professional Bachelor in Nursing	60	40.3
	University level Bachelor in Nursing	43	22.8
	Master in Nursing	9	6.0
Work experience	< 3 years	22	14.8
	3–5 years	30	20.1
	6–10 years	29	19.5
	> 10 years	68	45.6

129 (86.6%) of the sample were women. The largest proportion of study participants consisted of nurses with Diploma Nurse (46; 30.9%) and College level Professional Bachelor in Nursing (60; 40.3%) qualifications. Almost half (68; 45.6%) of nurses participating in the study had more than 10 years of work experience in anesthesia and intensive care.

### 2.3 Data collection

Data were collected in February–June 2024. The chosen data collection method was a questioning survey by using a closed-ended questionnaire. Quantitative research methods measure phenomena numerically and seek to ensure that the research results are reliable and objective (28).

#### 2.3.1 Research questionnaire

NASA Task Load Index (NASA-TLX) (32–34), The Practice Environment Scale of the Nursing Work Index (PES-NWI) (25, 26), and The Questionnaire of the Relation Between Job Satisfaction and Workload (Q-RJSW) (27) were used for data collection.

The first part of the questionnaire consisted of four sociodemographic questions (gender, age, education, work experience).

The second part of the questionnaire consisted of the Task Load Index (NASA-TLX), which is a subjective, multidimensional assessment tool that rates perceived workload in order to assess a task, system, or team effectiveness and task loading. This tool assesses aspects of workload which are important in healthcare (22, 24, 35). The Hart and Staveland (34) NASA-TLX tool assesses workload on five 7-point scales with increments of high, medium and low estimates for each point result in 21 gradations on the scales. NASA-TLX is divided into 6 subjective subscales: mental demand (how mentally demanding the task was), physical demand (how physically demanding the task was), temporal demand (how long it took to complete the task), performance (how well the task was performed), effort (how hard one had to work to achieve the result) and frustration (whether one felt insecure, frustrated, irritated, tense, and annoyed while performing the task).

The third part of the questionnaire consisted of the Practice Environment Scale of the Nursing Work Index (PES-NWI) (36, 37). The purpose of the scale is to identify factors that are important for nurses’ job satisfaction by assessing aspects of the professional environment that can positively or negatively affect nursing practice. The PES-NWI measures the nursing practice environment factors that enhance a nurse’s ability to practice nursing skillfully by delivering high quality care (36–38). The questionnaire focuses on nurses’ participation in hospital affairs, nursing basics for quality care, nursing administrator’s skills of leadership and support for nurses, staff and resource adequacy, and professional relationships between nurses and physicians (25).

The fourth part of the tool is the scale on Relation Between Job Satisfaction and Workload (Q-RJSW) (26–28). The questions reflect nurses’ job satisfaction, motivation, relationships with other employees, professional development opportunities, recognition from management/administration staff, appraisal processes, job stability, and compensation (26–28).

#### 2.3.2 Linguistic adaptation

Linguistic adaptation of the components of the research questionnaire [NASA Task Load Index (NASA-TLX), The Practice Environment Scale of the Nursing Work Index (PES-NWI), and The Questionnaire of the Relation Between Job Satisfaction and Workload (Q-RJSW)] was performed. It consisted of the following steps (39–42):

- Permission from the developers of the questionnaire parts: All formal permissions to use the aforementioned research instruments, which constitute the second to fourth parts of the research instrument in this study, were obtained from the developers of the instruments.
- Forward translation: The questionnaire was converted from the original English language to the target Lithuanian language while maintaining the original meaning and intent. The translation was performed by one professional translator representing a formal certified translation agency in Lithuania and one translator from Higher education institution of Lithuania. After the translation, two translated versions were obtained and a panel discussion was then organized with both translators, four anesthesia and intensive care nurses, and both authors of the article. The translators read each other’s translated instruments into Lithuanian and documented recommendations for improvement. Each translation version was read by four anesthesia and intensive care nurse and both authors of the article. All the panel participants documented their recommendations and comments on the translation, and a review document for each version was provided to both translators via email. However, remarks were also communicated verbally during this live meeting phase. The translators took into account the comments and collaborated in order to develop a single version of the research instrument, which was sent to all panel participants. Another online meeting of the entire panel was organized, during which one single version of the questionnaire was read, corrections were made, and consensus was reached on the final version of the questionnaire in Lithuanian.
- Backward translation: The adapted questionnaire in Lithuanian was translated back to the original English language to verify the accuracy and consistency of the translation. This translation from Lithuanian to English was already done by two other translators, but the principle remained the same - one translator represented a formal certified professional translation agency in Lithuania, and the other - a Lithuanian higher education institution. With two translation versions in English available, an online panel discussion was organized, during which comments and recommendations were provided to the translators of the forward translation stage. These translators improved the questionnaire and formed the pre-final single translated version in Lithuanian.
- Pretesting: The adapted questionnaire in Lithuanian was administered to a small sample of the target population consisting of three anesthesia and intensive care nurses (these nurses did not participate in the translation process panels) in order to identify any areas of confusion, ambiguity, or cultural insensitivity. Each expert nurse documented own comments and recommendations. The questionnaire was improved based on them. The Lithuanian version developed at this stage was forwarded to the translators and panelists from all stages for final comments, and a consensus was reached on the final version of the questionnaire, which was used in this empirical study.

#### 2.3.3 Reliability

The internal consistency reliability of the scales was calculated: experience in performing tasks at work scale (6 items, Cronbach’s *α* = 0.770); satisfaction with the professional environment scale (31 items, Cronbach’s *α* = 0.892); job satisfaction scale (20 items; Cronbach’s *α* = 0.868); total questionnaire (57 items; Cronbach’s *α* = 0.914).

#### 2.3.4 Validity

The face validity procedure was used to assess the validity of the research questionnaire. Two experts were invited to examine the research questionnaire for face validity (43). Inclusion criterion for experts was the following: nurse practitioner with a master’s degree in nursing who have experience not less than 10 years in anesthesia and intensive care nursing. Both experts were women, one with 15 years of practical experience and the other with 18. Both nurses have completed bachelor’s and master’s degrees in nursing at university level, are engaged in practical work, and are active in mentoring.

The experts were given an expert appointment letter for a face validity form for evaluation. The research tool together with face validity evaluation form was distributed through email together with a cover letter, synopsis of the research and declaration form for each appointed expert. The expert panel came with clear instructions on the research tool evaluation and the completed evaluation form was returned to researchers through the email.

The research tool evaluation criteria were the following (44): (i) use of correct and appropriate grammar; (ii) adjusted use of appropriate language; (iii) use of correct spelling; (iv) correct sentence structure; (v) appropriate writing size; (vi) appropriate format; (vii) appropriate content. Two values - Yes (agree) and No (disagree) - were used to rate the research instrument against each of the seven criteria. Experts were asked to provide comments and suggestions to improve the research questionnaire.

The Cohen Kappa Coefficient (CK) was applied to the face validity procedure. CK is a statistical measure used to assess the level of agreement between two or more raters when categorizing data. While primarily used for inter-rater reliability, it can be adapted to evaluate face validity by assessing the agreement among experts regarding the appropriateness of a measurement tool or its items (44). In the context of face validity, CK helps determine if the measurement tool appears to be measuring what it’s intended to measure.

CK is calculated to quantify the level of agreement between the raters. A high CK value (typically above 0.60 or 0.70) indicates a good level of agreement among the experts, suggesting that the measurement tool has good face validity. CK value equal to +1 indicates complete agreement between the two evaluators, while–1 indicates complete disagreement (45). If CK estimates a value of 0, then it shows that there is no correlation between the evaluations of the two evaluators. Any agreement and disagreement are sole. Average percentage of agreement was 85.7%, which indicates the strong level of agreement (43).

CK is calculated using the following formula: Kappa = (Po - Pe) / (1 - Pe)

Where: Po - the observed proportion of agreement (the proportion of items where experts agree); Pe - the expected proportion of agreement by chance (the probability that experts would agree by random chance).

Validity values were analyzed through seven evaluation criteria that were given to two experts. Validity values have been calculated by using Cohen Kappa calculation through SPSS for Windows 29.0 software. The Kappa Coefficient value shows a value of 0.857 (*p* = 0.001 < alpha = 0.05) where it is at a good level of validity and suitable to be used to collect research data (46). All comments and suggestions from experts were taken and the researcher made corrections and improvements to the research questionnaire.

### 2.4 Data analysis

SPSS for Windows 29.0 was used for data analysis. The Kolmogorov and Smirnov test was used to assess whether continuous variables were normally distributed. All continuous data were normally distributed, therefore, in descriptive statistics, data are presented as mean and standard deviation (SD). The Student’s *t*-test was used to test statistical hypotheses between two independent samples; while comparing three or more independent samples, one-way analysis of variance (ANOVA) was applied. All qualitative variables analyzed in the study were ordinal, therefore, non-parametric criteria were used to test statistical hypotheses. Cross-tabulations were created to compare two independent samples, and the *χ*^2^ test was used to assess relationships between groups. Pearson’s linear correlation coefficient (*r*) was used to assess linear relationships between job satisfaction, work in a team and collaborative professional environment, experiences in the professional environment, subjective workload, and socio-demographic indicators. Rank-order logistic regression analysis was applied for the clarification of complex relationships. While testing statistical hypotheses, results were considered statistically significant when the *p* < 0.05 (27).

## 3 Results

Results are structured into four key areas: (i) nurses’ assessment of subjective workload, professional environment and job satisfaction; (ii) relationships between nurses’ subjective workload, working in a team and collaborative professional environment, satisfaction with the professional environment, and job satisfaction; (iii) the relationship between nurses’ professional environment and job satisfaction; (iv) complex relationships.

### 3.1 Nurses’ assessment of subjective workload, professional environment and job satisfaction

The results showed that 56 (37.6%) nurses rated their workload as high, the majority of respondents (121; 81.2%) rated their professional environment as moderately satisfactory and a few respondents (6; 4.0%) experienced low job satisfaction (see Tables 2–4).

**Table 2:** Assessment of subjective workload (*N* = 149).

Values	Subjective workload *n*/%
Very low	11 (7.4)
Low	29 (19.4)
Moderate	53 (35.6)
High	39 (26.2)
Very high	17 (11.4)

**Table 3:** Assessment of professional environment (*N* = 149).

Values	Professional environment *n*/%
Low	12 (8.1)
Moderate	121 (81.2)
High	16 (10.7)

**Table 4:** Assessment of job satisfaction (*N* = 149).

Values	Job satisfaction *n*/%
Low	33 (22.1)
Moderate	110 (73.8)
High	6 (4.0)

When assessing the subjective workload of nurses, they were divided into quartiles according to the total possible score (0 – minimum workload, 24 – maximum workload) in the second part of the survey tool (questions 5–10). The results showed that 53 (35.6%) respondents rated their subjective workload as average, and 56 (37.6%) respondents rated their subjective workload as high to very high (see Table 2).

When assessing nurses’ experiences in the professional environment, the study participants were divided into tertiles according to the maximum possible score (31 – the minimum possible score, which corresponded to maximum satisfaction with the professional environment, 124 – the maximum possible score, which corresponded to minimum satisfaction with the professional environment) in the third part of the questionnaire (questions 11–41). The results showed that the majority of study participants (121; 81.2%) assessed the professional environment as average (see Table 3).

When assessing respondents’ job satisfaction, the study participants were divided into tertiles according to the maximum possible score (0 – maximum job satisfaction, 80 – minimum job satisfaction) in the fourth part of the survey questionnaire (questions 42–61). The results showed that a large proportion of nurses are moderately (110; 73.8%) satisfied with their job (see Table 4).

In summary, it can be stated that all three variables - subjective workload, professional environment, and job satisfaction - are rated as average by the majority of nurses. So the average of experienced subjective workload was 12.92 (5.366) out of 24 possible points, the average satisfaction with the professional environment was 73.52 (12.440) out of 124 possible points, and job satisfaction was 35.16 (11.056) out of 80 possible points.

Nurses’ subjective assessment of workload differed statistically significantly among study participants when divided by age group. Younger nurses assessed their workload and job satisfaction as higher than older nurses. Nurses’ job satisfaction differed statistically significantly among respondents by age group. Older nurses were more satisfied with their jobs than the younger. Job satisfaction did not differ statistically significantly among study participants (see Table 5).

**Table 5:** Statistical differences between groups while assessing subjective workload, professional environment and job satisfaction (*N* = 149).

Sociodemogra-phic indicators	Groups	Subjective workload		Professional environment		Job satisfaction	
		Mean (SN)	*p* – value	Mean (SN)	*p* – value	Mean (SN)	*p* – value
Gender	Man	13.40 (5.276)	0.668	74.70 (9.603)	0.649	38.80 (10.263)	0.114
	Woman	12.84 (5.396)		73.33 (12.845)		34.60 (11.105)	
Age	20–30 years old	14.85 (4.710)	**0.012**	75.65 (15.155)	0.182	35.75 (11.506)	**0.030**
	31–40 years old	12.96 (5.133)		74.57 (12.719)		38.11 (11.069)	
	≥ 41 years old	11.65 (5.631)		71.34 (9.899)		32.55 (10.281)	
Education level	Diploma Nurse	11.70 (5.962)	0.242	72.33 (10.946)	0.165	32.13 (10.776)	0.054
	College level Professional Bachelor in Nursing	13.18 (5.111)		72.42 (14.520)		37.20 (11.483)	
	University level Bachelor in Nursing	13.62 (5.257)		74.94 (8.842)		34.32 (10.197)	
	Master in Nursing	14.78 (5.366)		81.56 (14.604)		40.22 (9.550)	
Work experience	< 3 years	12.82 (6.359)	0.93	69.55 (10.751)	0.153	32.45 (8.040)	0.159
	3–5 years	14.97 (4.867)		76.63 (10.565)		37.87 (12.931)	
	6–10 years	13.03 (4.066)		75.52 (17.435)		37.38 (11.422)	
	> 10 years	12.00 (5.569)		72.57 (10.869)		33.90 (10.651)	

Statistically significant results are marked in bold (Student’s t-test / one-way ANOVA).

No difference was found in the sample of nurses by gender and age (*χ*^2^ = 2.943, *p* = 0.23). No difference was found among anesthesia and intensive care nurses by gender and education (*χ*^2^ = 3.348, *p* = 0.341). No difference was found among anesthesia and intensive care nurses by gender and work experience in this field (chi square = 3.467, *p* = 0.325).

To assess how subjective workload is related to socio-demographic, a rank-order logistic regression analysis was used. The continuous variable “subjective workload” (the total possible score from 5–10 questions in the survey tool) was recoded into a categorical variable, which took on values from 1 – very low workload to 5 – very high workload. However, when assessing whether we had a sufficient number of subjects classified as “very low” or “very high,” it was found that only 7.4 and 11.4 percent of them were so, so we recoded the variable “subjective workload” to take on only three possible categories: 1 – low workload, 2 – medium workload, 3 – high workload. In the final linear regression model, we used the variable “gender” as a factor, and the variables “age” and “education” as covariates (the covariate “experience” was not used in the final model because it reduced the reliability of the model).

A ordinal logistic regression analysis was used for assessing the dependence of workload on sociodemographic indicators. While assessing the subjective workload whether it is a sufficient number of subjects classified as “very low”/“very high,” it was determined 7.4 and 11.4% of such subjects.

In the final ordinal regression model the assumption of parallelism of the lines was satisfied (*χ*^2^ = 4.231; df = 3; *p* = 0.238). The likelihood ratio criterion (*χ*^2^ = 8.877; df = 3; *p* = 0.031) and the Pearson (*χ*^2^ = 41.872; df = 37; *p* = 0.268) and deviance (*χ*^2^ = 47.445; df = 37; *p* = 0.117) criteria indicated a sufficiently good suitability of the model to the data; Nagelkerke R^2^ = 0.065. The model correctly classified 40% of cases when “subjective workload” = 1, 22.6% of cases when “subjective workload” = 2 and 66.1% of cases when “subjective workload” = 3. Therefore, sociodemographic indicators (gender, age, education) had limited relationship to the perception of subjective workload (see Table 6).

**Table 6:** Results of the ordinal logistic regression model ‘Subjective workload’* (*N* = 149).

Independent variable (predictor)	*β*	SE	Wald *χ*^2^	df	*p*	95% PI (lower – upper)
Age	−0.486	0.211	5.32	1	0.021	−0.898 – −0.073
Education	0.145	0.191	0.578	1	0.447	−0.229 – 0.520
Gender (0 = Male)	0.06	0.451	0.017	1	0.895	−0.825 – 0.944

*Model suitability: *χ*^2^(3) = 8.877, *p* = 0.031; Pseudo *R*^2^: Cox & Snell = 0.058, Nagelkerke = 0.065, McFadden = 0.027.

In the final ordinal logistic regression model the assumption of parallelism of the lines was met (*χ*^2^ = 2.024; df = 4; *p* = 0.555). A sufficiently good fit of the model to the data is indicated by the likelihood ratio criterion (*χ*^2^ = 44.324; df = 3; *p* = 0.005) and the Pearson (*χ*^2^ = 27.205; df = 37; *p* = 0.708) and deviance (*χ*^2^ = 27.300; df = 37; *p* = 0.658) criteria; Nagelkerke *R*^2^ = 0.272. The model correctly classified 51% of cases when “job satisfaction” = 1.37.2% of cases when “job satisfaction” = 2 and 73.4% of cases when “job satisfaction” = 3. Sociodemographic indicators (age, gender, education, work experience), have some influence on job satisfaction, but explain only part of its variation (see Table 7).

**Table 7:** Results of the ordinal regression model ‘Job satisfaction’* (*N* = 149).

Independent variable (predictor)	*β*	SE	Wald *χ*^2^	df	*p*	95% PI (lower – upper)
Age	−0.298	0.351	0.722	1	0.396	−0.985 – 0.389
Education	−0.143	0.229	0.388	1	0.533	−0.592 – 0.307
Work experience	0.146	0.238	0.376	1	0.54	−0.321 – 0.613
Gender (0 = Male)	0.454	0.58	0.613	1	0.434	−0.683 – 1.592

*Model suitability: *χ*^2^(3) = 44.324, *p* = 0.005; Pseudo *R*^2^: Cox & Snell = 0.241, Nagelkerke = 0.272, McFadden = 0.071.

Anesthesia and intensive care nurses experience above average or high subjective workload. Those nurses who experience high workload experience lower job satisfaction than those who experience lower workload. Similarly, nurses who evaluate their professional environment positively experience lower workload. Therefore, high workload reduces satisfaction, while a favorable environment reduces perceived workload.

Pearson r was used to assess the direction and strength of the relationships between sociodemographic indicators and subjective workload, satisfaction with the professional environment and job satisfaction. A negative correlation was found between the age of nurses and subjective workload – older nurses experienced lower subjective workload (*r* = − 0.241; *p* = 0.03) (see Table 8).

**Table 8:** Correlations between sociodemographic indicators and subjective workload, satisfaction with the professional environment and job satisfaction (*N* = 149).

Variable	Pearson *r*; *p* – value*			
	Gender	Age	Education	Work experience
Subjective workload	*r* **=** −0.035; *p* = 0.668	*r* = **−0.241;** *p* = **0.003**	*r* = **0.162;** *p* = **0.049**	*r* = −0.134; *p* = 0.102
Professional environment	*r* = −0.038; *p* = 0.649	*r* = −0.147; *p* = 0.075	*r* = 0.152; *p* = 0.064	*r* = 0.008; *p* = 0.920
Job satisfaction	*r* = −0.130; *p* = 0.114	*r* = −0.139; *p* = 0.091	*r* = 0.140; *p* = 0.089	*r* = −0.026; *p* = 0.751

Statistically significant results are marked in bold.

A positive correlation was also found between the education and subjective workload – nurses with a higher level of education experienced higher subjective workload (*r* = 0.162; *p* = 0.049). No other statistically significant relationships were found.

### 3.2 Relationships between nurses’ subjective workload, working in a team and collaborative professional environment, satisfaction with the professional environment, and job satisfaction

Pearson *r* coefficient was used to analyze the relationships between subjective workload, teamwork and collaborative work environment, satisfaction with the work environment, and job satisfaction. A positive, statistically significant correlation was found between all variables.

Nurses who experienced higher subjective workload felt lower satisfaction with the professional environment (*r* = 0.306; *p* < 0.01) and lower job satisfaction (*r* = 0.199; *p* = 0.015). Conversely, nurses who experienced lower subjective workload felt higher satisfaction with the professional environment and higher job satisfaction. The relationship between subjective workload and satisfaction with the professional environment is weak (*r* = 0.306; *p* < 0.001), and between subjective workload and job satisfaction is very weak also (*r* = 0.199; *p* = 0.015). Nurses who felt more satisfaction with the professional environment felt more job satisfaction; correlation between job satisfaction and professional environment is moderate (*r* = 0.578; *p* < 0.001). Nurses who worked in a team-based and collaborative professional environment experienced greater job satisfaction; correlation between these two variables is weak (*r* = 0.365; *p* < 0.001) (see Table 9).

**Table 9:** Correlations between subjective workload, job satisfaction, professional environment, and teamwork and collaborative professional environment (*N* = 149).

		Job satisfaction	Professional environment	Teamwork and collaborative work environment
Subjective workload	Pearson Correlation	0.199*	0.306**	-
	Sig. (2-tailed)/ *p* – value	0.015	<0.001	-
Job satisfaction	Pearson Correlation	1	0.578**	0.365**
	Sig. (2-tailed)/ *p* – value	-	<0.001	<0.001

*p ≤ 0.05; **p ≤ 0.01.

Anesthesia and intensive care nurses have an average assessment of their professional environment. Nurses working in a favorable professional environment experience lower workload and feel higher job satisfaction than those working in unfavorable environments. This means that a positively assessed professional environment for nurses reduces workload and increases job satisfaction.

### 3.3 The relationship between nurses’ professional environment and job satisfaction

It was found that anesthesia and intensive care nurses who rated their workload as average and evaluated their professional environment positively rated their job satisfaction at an average of 27.32 (11.395) points, while nurses who felt high or low workload and evaluated their professional environment negatively rated their job satisfaction at an average of 36.52 (10.457) points. The difference in means was statistically significant (Student’s t = 3.760, df = 147, *p* < 0.001), the difference in means was 9.202 (95% confidence interval (CI) 4.366–14.037). This indicates that the nurses who assessed their subjective workload as average and their professional environment as positive felt greater satisfaction with their work (see Table 10).

**Table 10:** Relationships between workload, professional environment and job satisfaction (*N* = 149).

Workload and professional environment	Mean of job satisfaction	Standard deviation/SD
Moderate workload and positive environment	27.32	11.395
High or low workload and negative environment	36.52	10.457

Job satisfaction among anesthesia and intensive care nurses is average. The highest satisfaction is felt by those nurses who work in a supportive professional environment and experience average workload.

### 3.4 Complex relationships

One-way analysis of variance was used to determine the relationship between managerial support and subjective workload with job satisfaction: nurses who received adequate managerial support and rated their workload as low (group 1) rated their job satisfaction on average 30.96 (10.114) points; nurses who received adequate managerial support and rated their workload as high (group 2) rated their job satisfaction on average 34.07 (10.619) points; nurses who received low managerial support and rated their workload as low (group 3) assessed their job satisfaction on average 40.05 (10.745) points; nurses who received low support from their managers and rated their workload as high (group 4) had an average job satisfaction score of 40.56 (10.311). A one-way analysis of variance showed that the mean job satisfaction score differed statistically significantly between at least two study groups (*F*(3.145) = [7.580], *p* < 0.001).

Tukey’s HSD test showed that the difference between nurses who received adequate support from their managers and rated their workload as low and nurses who received adequate support from their managers and rated their workload as high was statistically insignificant (*p* = 0.470, 95% CI (−)8.68–2.46).

Anesthesia and intensive care nurses who receive adequate support from their managers experience greater job satisfaction, regardless of workload (see Table 9).

Nurses who rated their workload as low and evaluated their professional environment positively rated their job satisfaction at an average of 32.44 (9.425) points, while nurses who felt a high workload and evaluated their professional environment negatively rated their job satisfaction at an average of 36.13 (11.464) points (see Table 10).

The difference in means was statistically significant (Student’s t = 1.805, df = 147, *p* = 0.037), the difference in means – 3.691 (95% CI 0.350–7.733). This indicated that nurses who assessed their workload as low and assessed their professional environment positively felt greater satisfaction with their work, which confirms the hypothesis that in the presence of a high workload and an unfavorable professional environment, the job satisfaction of anesthesia and intensive care nurses decreases.

Subjective workload, work environment, and job satisfaction of anesthesia and intensive care nurses are interrelated and influence each other. Nurses who work in an unfavorable environment without managerial support, collaboration, and teamwork experience higher workload and lower job satisfaction. This means that job satisfaction decreases when there is a high workload and an unfavorable work environment (Tables 11–13).

**Table 11:** The relationship between managerial support, workload, and job satisfaction.

Managerial support and workload	Mean of job satisfaction	Standard deviation/SD
Adequate management support and low workload	30.96	10.114
Adequate management support and heavy workload	34.07	10.619
Low management support and low workload	40.05	10.745
Low management support and high workload	40.56	10.311

**Table 12:** The relationship between workload, professional environment and job satisfaction.

Workload and professional environment	Mean of job satisfaction	Standard deviation/SD
Low stress and positive environment	32.44	9.425
High workload and negative environment	36.13	11.464

**Table 13:** Summary of correlations between key variables.

Pair of variables	*r*	*p-*value	Significance
Workload – Job Satisfaction	0.199	0.015	* (*p* < 0.05)
Workload – Satisfaction with the work environment	0.306	<0.001	* (*p* < 0.01)
Satisfaction with the work environment – Workload	0.578	<0.001	* (*p* < 0.01)
Job satisfaction – Team-based and collaborative work environment	0.365	<0.001	** (*p* < 0.01)

*p ≤ 0.05; **p ≤ 0.01; ***p ≤ 0.001.

## 4 Discussion

Anesthesia and intensive care nurses face a variety of workload and professional environment challenges, but there has been insufficient research into how job satisfaction may relate to and be related to workload and professional environment. Therefore, the study aimed to determine the relationship between the workload, professional environment and job satisfaction of anesthesia and intensive care nurses, i.e., to determine the subjective workload of anesthesia and intensive care nurses; to assess the professional environment of anesthesia and intensive care nurses; to assess the job satisfaction of anesthesia and intensive care nurses; to determine the relationship between the subjective workload, professional environment and job satisfaction of anesthesia and intensive care nurses.

The results of the study revealed that anesthesia and intensive care nurses who experience higher subjective workload experience lower job satisfaction. This relationship is consistent with the results of a study by Hellín et al. (18), who found that nurses in Spain experience high workload, which leads to physical and emotional exhaustion, higher stress levels and dissatisfaction with organizational management. Similar results were obtained in a study conducted by Tomaszewska et al. (12) who found in Poland that increased workload, resulting from a lack of time to complete tasks at work, reduces job satisfaction. Nakweenda et al. (1) in Africa found that higher workload and an unfavorable professional environment directly affect job satisfaction. This shows that regardless of geographical boundaries, anesthesia and intensive care nurses everywhere face a professional environment that is dependent on workload and can directly affect job satisfaction.

The relationship between high workload and satisfaction, regardless of the country or geographical location, is significant and affects anesthesia and intensive care nurses negatively. This is largely due to the inherent stress and demanding nature of these specialized nursing roles, which are often accompanied by emotional strain, burnout (47). It can also be assumed that anesthesia and intensive care nurses work in high-pressure environments with constant exposure to critical situations, suffering, and death, leading to significant emotional and psychological strain (12). The combination of high stress, emotional strain, and heavy workload significantly increases the risk of burnout among anesthesia and intensive care nurses, impacting their mental and physical health; burnout, in turn, leads to reduced job satisfaction, decreased motivation (18). However, while geographical location might influence specific stressors (e.g., staffing levels, access to resources), the core issues of high workload, emotional strain, and burnout are generally consistent across different locations (48).

Bolado et al. (21) conducted the study in Southern Ethiopia with the aim to determine the relationship between job satisfaction and professional environment. It was found that higher workload was significantly associated with moderate stress, poor professional environment further increases stress, which in turn reduces job satisfaction. A study conducted in the Netherlands highlighted that workload have less direct significance for satisfaction, but that the professional environment and intrinsic motivation for the nursing profession are of great importance (9). Comparing the results of the study in Lithuania, Ethiopia and the Netherlands, it can be stated that regardless of the geographical location or the level of development of the healthcare system, the job satisfaction of anesthesia and intensive care nurses depends most on the environment, which negatively affects job satisfaction, and high workload contributes to a decrease in job satisfaction. The obtained research results allow us to boldly state that A negative work environment and high workload consistently contribute to decreased job satisfaction for these nurses. The work environment encompasses various aspects of the workplace, including physical conditions, interpersonal relationships, organizational culture, and the overall atmosphere (49). A positive work environment, characterized by supportive colleagues, effective leadership, adequate resources, and a healthy atmosphere, fosters job satisfaction. Conversely, a negative work environment with factors like poor communication, lack of teamwork, inadequate resources, and high stress levels can lead to dissatisfaction (50). This internationally obtained empirical evidence suggests that our study has the potential to be expanded by including more variables and looking for relationships between them, while maintaining a focus on the job satisfaction of anesthesia and intensive care nurses.

A study conducted in Saudi Arabia showed that managers’ abilities, leadership, and support for nurses were significantly correlated with overall job satisfaction (23). Our study also revealed that managerial support is one of the most important determinants of job satisfaction for anesthesia and intensive care nurses worldwide, regardless of workload. Managerial support is indeed a significant factor influencing job satisfaction for anesthesia and intensive care nurses, often outweighing the impact of workload. While workload is a factor, supportive management practices, such as clear communication, recognition, and opportunities for professional development, contribute more to overall job satisfaction in these demanding roles (51). Strong leadership, guidance, and support from nurse managers create a positive work environment that contributes to higher job satisfaction among anesthesia and intensive care nurses. This support can manifest as employee-oriented leadership, open communication, and actively addressing concerns (52).

Wising et al. (53) conducted a study in Sweden which showed that nurses working in an environment where collaboration is based on mutual trust and responsibility feel more satisfied with their work and are not afraid to demonstrate their competence and responsibility for their actions in teamwork. From both studies, conducted in Lithuania and Sweden, it is evident that collaboration and teamwork between anesthesia and intensive care nurses and physicians anesthetists is one of the key factors determining nurses’ job satisfaction and sense of professionalism. Effective teamwork and collaboration between anesthesia and intensive care nurses and physicians is crucial for job satisfaction and a strong sense of professionalism among nurses, according to nursing and medical research. This collaboration fosters a positive work environment, improves communication, and enhances patient care (54). When nurses experience positive collaboration with their physician colleagues, their job satisfaction tends to increase. This satisfaction stems from a sense of shared responsibility, mutual respect, and effective communication (55).

A study conducted in United Kingdom found that high job demands lead to stress and frustration, then nurses feel isolated (16). A study from Indonesia revealed that higher psychological workload significantly affects nurses’ stress, which negatively affects their job satisfaction (2). These results are consistent with the results of our study. Hence, it can be stated that high workload, unfavorable professional environment and psychological stress significantly reduce anesthesia and intensive care nurses’ job satisfaction, and these factors can occur regardless of geographical location or characteristics of the healthcare system. These factors are often independent of geographical location or the specific healthcare system. While the details may vary, the fundamental challenges of high workload, poor environment, and psychological stress negatively impact anesthesia and intensive care nurses across different settings (56).

The limitation of the study is that subjective workload, professional environment and job satisfaction are defined according to specific variables. Although many more variables that may influence these phenomena are found in the literature, quantitative research allows the analysis of only certain predetermined aspects. The limitation of quantitative research arises from its structure. It allows the measurement and analysis of pre-selected variables, but is unable to fully reveal the more complex and subjective experiences and contexts that can be found in qualitative research (57). Quantitative research allows for accurate assessment of relationships and trends between certain variables in a larger population of subjects, but limits the depth of understanding of phenomena.

## 5 Conclusion

There is a relationship between anesthesia and intensive care nurses’ workload, professional environment and job satisfaction. Nurses who experience a high workload experience lower job satisfaction than those who experience a lower workload. Likewise, nurses who evaluate their professional environment positively experience lower workloads.

Anesthesia and intensive care nurses working in a supportive professional environment experience lower workload and greater job satisfaction than those working in an unfavorable environment. This means that a positively perceived professional environment for nurses reduces workload and increases job satisfaction. The greatest satisfaction is felt by those anesthesia and intensive care nurses who work in a supportive professional environment and experience an average workload.

The subjective workload, professional environment and job satisfaction of anesthesia and intensive care nurses are interrelated and influence each other. Nurses who work in an unfavorable environment without managerial support, cooperation and teamwork experience higher workload and feel lower job satisfaction. This means that job satisfaction decreases when there is a high workload and an unfavorable professional environment.

Therefore, in the future, it would be useful to conduct a qualitative study that would allow for a deeper understanding of the experiences of anesthesia and intensive care nurses, based on their personal experiences and opinions. A qualitative approach would provide a broader and deeper understanding of subjective workload, professional environment and job satisfaction, including the individual experiences of anesthesia and intensive care nurses.

## Data Availability

The raw data supporting the conclusions of this article will be made available by the authors, without undue reservation.
